# Reduced *BRCA1* transcript levels in freshly isolated blood leukocytes from *BRCA1* mutation carriers is mutation specific

**DOI:** 10.1186/s13058-016-0739-8

**Published:** 2016-08-17

**Authors:** Rania Chehade, Rachael Pettapiece-Phillips, Leonardo Salmena, Max Kotlyar, Igor Jurisica, Steven A. Narod, Mohammad R. Akbari, Joanne Kotsopoulos

**Affiliations:** 1 Women’s College Research Institute, Women’s College Hospital, 76 Grenville Street, 6th Floor, Toronto, ON Canada; 2 Faculty of Medicine and Dentistry, University of Alberta, 2J2.00 WC Mackenzie Health Sciences Centre, Edmonton, AB Canada; 3 Department of Nutritional Sciences, University of Toronto, FitzGerald Building, 150 College Street, Room 316, Toronto, ON Canada; 4 Princess Margaret Cancer Centre, University Health Network, Toronto, ON Canada; 5 Department of Pharmacology and Toxicology, University of Toronto, Toronto, ON Canada; 6 Department of Medical Biophysics and Computer Science, University of Toronto, Toronto, ON Canada; 7 Dalla Lana School of Public Health, University of Toronto, 155 College Street Health Science Building, 6th Floor, Toronto, ON Canada

**Keywords:** BRCA1, mRNA expression, Hereditary breast and ovarian cancer, Haploinsufficiency

## Abstract

**Background:**

*BRCA1* mutation carriers face a high lifetime risk of developing both breast and ovarian cancer. Haploinsufficiency is thought to predispose these women to cancer by reducing the pool of available BRCA1 transcript and protein, thereby compromising BRCA1 function. Whether or not cancer-free *BRCA1* mutation carriers have lower messenger (m)RNA transcript levels in peripheral blood leukocytes has not been evaluated. The primary aim of this study was to characterize an association between *BRCA1* mutation status and *BRCA1* mRNA leukocyte expression levels among healthy women with a *BRCA1* mutation.

**Method:**

RNA was extracted from freshly isolated peripheral blood leukocytes of 58 cancer-free, female participants (22 *BRCA1* mutation carriers and 36 non-carriers). The expression levels of 236 cancer-associated genes, including *BRCA1,* were quantified using the Human Cancer Reference gene panel from the Nanostring Technologies nCounter Analysis System.

**Results:**

Multivariate modeling demonstrated that carrying a *BRCA1* mutation was the most significant predictor of *BRCA1* mRNA levels. *BRCA1* mRNA levels were significantly lower in *BRCA1* mutation carriers compared to non-carriers (146.7 counts vs. 175.1 counts; *P* = 0.002). Samples with *BRCA1* mutations within exon 11 had lower *BRCA1* mRNA levels than samples with mutations within the 5′ and 3′ regions of the *BRCA1* gene (122.1 counts vs. 138.9 and 168.6 counts, respectively; *P* = 0.003). Unsupervised hierarchical clustering of gene expression profiles from freshly isolated blood leukocytes revealed that *BRCA1* mutation carriers cluster more closely with other *BRCA1* mutation carriers than with *BRCA1* wild-type samples. Moreover, a set of 17 genes (including *BRCA1*) previously shown to be involved in carcinogenesis, were differentially expressed between *BRCA1* mutation carriers and non-carriers.

**Conclusion:**

Overall, these findings support the concept of *BRCA1* haploinsufficiency wherein a specific mutation results in dosage-dependent alteration of *BRCA1* at the transcriptional level. This study is the first to show a decrease in *BRCA1* mRNA expression in freshly isolated blood leukocytes from healthy, unaffected *BRCA1* mutation carriers.

**Electronic supplementary material:**

The online version of this article (doi:10.1186/s13058-016-0739-8) contains supplementary material, which is available to authorized users.

## Background

Women with a mutation in the breast cancer susceptibility gene 1 (*BRCA1*) face a high lifetime risk of developing breast and ovarian cancer estimated to be as high as 80 % and 40 %, respectively [1–4]. *BRCA1* regulates several key functions pertinent to cell survival, proliferation, and differentiation [5, 6]. In particular, *BRCA1* helps maintain genomic stability by participating in the cellular DNA damage response through homologous recombination (HR)-mediated repair of double-stranded DNA breaks (DSBs) [7]. There is accumulating evidence that *BRCA1* haploinsufficiency is a driver of tumor predisposing events in *BRCA1* mutation carriers [8].

For haploinsufficiency to be an early driver of *BRCA1*-associated cancer development, mutation-dependent reduction in BRCA1 expression levels should be associated with a loss of function [8–10]. Most studies that have characterized phenotypic alterations suggest that *BRCA1* heterozygous cells have reduced functions in DNA damage repair, hormonal regulation, cell fate changes, transcriptional regulation and autophagy [11–21]; however, little is known about whether the abrogated functions observed in *BRCA1* heterozygous cells are correlated with changes in BRCA1 transcript or protein levels [19, 22–24]. This is important in light of data suggesting that the type and location of a mutation can stratify cancer risk (i.e., breast vs. ovary), and the response to treatment [25–29].

Regulation of *BRCA1* gene expression is influenced by genetic and epigenetic mechanisms, and environmental factors such as genotoxic, hormonal, and metabolic stressors [30]. Understanding the contribution of the mutation status to basal expression levels of the *BRCA1* gene is a crucial step to delineating *BRCA1* haploinsufficiency. Previous studies using immortalized lymphoblastoid cell lines have reported differential messenger RNA (mRNA) or protein expression in *BRCA1* mutation carriers compared to non-carriers, suggesting a mutation-specific dosage effect [19, 24, 31]. In contrast, Feilotter et al. [18] did not find *BRCA1* to be among the set of 43 genes that can predict *BRCA1* mutation status by gene expression profiling. However, differences in *BRCA1* mRNA expression may have been masked by the continuous proliferative state of immortalized lymphoblastoid cell lines used in these experiments [22, 32–37]. There are no studies, to our knowledge, that have evaluated *BRCA1* transcript levels in freshly isolated blood leukocytes.

Notably, reduced BRCA1 protein expression in both inherited and sporadic forms of breast and ovarian cancer has been associated with a significant reduction in the levels of *BRCA1* mRNA, thereby supporting the utility of *BRCA1* transcript levels as a surrogate marker of BRCA1 function [38–40]. The overall goal of the current study was to evaluate the relationship between *BRCA1* mutation status (and mutation type) and mRNA expression among women with and without a *BRCA1* mutation, by studying freshly isolated blood leukocytes.

## Methods

### Study design and population

There were 58 women enrolled in the current study: 22 *BRCA1* mutation carriers and 36 non-carriers. All women were 18 years of age or older, none had a personal history of cancer, and none were pregnant or breastfeeding. The first group included women with a *BRCA1* mutation, identified from an existing database at the Familial Breast Cancer Research Unit, Women’s College Research Institute (WCRI, Toronto, Canada) who were contacted by letter. The second group included women from the general population who were recruited using various methods such as posters, newsletters or social media. A 30-minute study appointment was then scheduled at the WCRI for all the eligible participants. This research received approval from the Research Ethics Board at the Women’s College Hospital (number 2012-0055-B). All women provided informed consent to participate in the study by signing the provided consent form.

### Data and biological sample collection

Study participants completed a questionnaire, which collected information on various exposures, including reproductive and lifestyle factors, medical history, and family history of cancer. Standardized procedures were used to collect measurements of weight (kg) and height (m) to calculate body mass index (BMI; kg/m^2^). A phlebotomist drew blood into two labeled EDTA-containing tubes (approximately 8 mL) by venipuncture. The samples were placed on ice and delivered immediately to the Women’s College Hospital research laboratory for RNA extraction.

### RNA isolation and quantification

RNA was isolated from one of the two EDTA tubes using the LeukoLOCK Total RNA Isolation System (Ambion, USA). This system is optimized for use with human blood and offers the isolation of total RNA from the leukocyte population [41]. In order to maximize RNA isolation yield, all samples were stabilized with RNALater® within 35 minutes of the blood draw. The nucleic acid content was quantified using the Nanodrop spectrophotometer (ThermoScientific). Total RNA quality and quantity was then determined using the Agilent 2100 Bioanalyzer (The Centre for Applied Genomics, Toronto, Canada). The resulting extracted RNA was stored at -80 °C until required for further analysis.

### nCounter NanoString gene expression profiling

The nCounter Analysis System (NanoString Technologies) was used to measure mRNA gene expression (expressed as counts) at the University Health Network (Toronto, Canada) [42] using the Human Cancer Reference Kit consisting of 236 cancer-related genes. Briefly, the nCounter Analysis System probe library contains two sequence-specific probes, the capture probe and the reporter probe, for each gene of interest. Probe pairs are mixed with total RNA in one hybridization reaction, and then the structures are imaged with the use of fluorescent microscopy. Expression is measured by counting the number of unique color tags within the gene-probe tripartite structures and is reported as counts, a direct measure of the number of RNA transcripts of each gene of interest.

Data acquisition and normalization was carried out using the nSolver Analysis software version 2.0 (NanoString Technologies). Positive and negative controls were used to check for background expression. Reference housekeeping gene normalization was then performed to adjust counts relative to probes that are not expected to vary between samples or replicates, allowing meaningful comparisons between samples. We chose the set of housekeeping genes recommended by Nanostring, which comprised the following genes: *CLTC* (clathrin, heavy chain), *GAPDH* (glyceraldehyde-3-phosphate dehydrogenase), *GUSB* (glucuronidase, beta), *HPRT1* (hypoxanthine phosphoribosyltransferase 1), *TUBB* (tubulin, beta class 1), and *PGK1* (phosphoglycerate kinase 1).

### Statistical analysis

Student’s *t* test was used to compare continuous variables in mutation carriers and non-carriers and the chi-square test was used to test for differences in categorical variables. The Shapiro-Wilk test was used to verify the normality of *BRCA1* mRNA expression. As expression of *BRCA1* was normally distributed (*P* = 0.26), the Pearson correlation coefficient (*ρ*) was used to evaluate the correlation between *BRCA1* mRNA expression, *BRCA1* mutation status and various reproductive and lifestyle factors. Linear regression was used to evaluate the relationship between *BRCA1* mutation status and mRNA expression, adjusting for significant predictors of *BRCA1* mRNA levels including parity (parous/nulliparous), breastfeeding (ever/never), and menopausal status (premenopausal/postmenopausal). A significance level of *P* <0.05 was used as the criterion for including variables in the multivariate model.

We assigned the *BRCA1* mutations into one of three mutation clusters reported to be differentially associated with the risk of breast vs. ovarian cancer: group 1 contained mutations in exons 1–10; group 2 contained mutations in exon 11; and group 3 contained mutations in exons 12–22 [28]. One-way analysis of variance (ANOVA) was used to compare mean *BRCA1* expression levels between the three mutation clusters.

Unsupervised hierarchical clustering was performed using Pearson-centered correlation metric with centroid linkage to categorize samples into homogenous groups based on similar levels of gene expression. Heat maps were generated using Java Treeview [43]. Student’s *t* test was used to identify genes expressed differentially between *BRCA1* mutation carriers and non-carriers by testing for differences in mean gene expression levels with a Benjamini-Hochberg false discovery rate of *P* < 0.05. The PathDIP database (http://ophid.utoronto.ca/pathDIP) was used to identify over-represented signaling pathways using data from significantly upregulated and downregulated genes through functional enrichment analysis. Statistical significance was defined at the level of *P* < 0.05 and all analyses were carried out using SPSS, IBM® SPSS® Statistics, version 23, 2015.

## Results

### Characteristics of study participants

Characteristics of the study subjects are provided in Table 1. Women with a *BRCA1* mutation were significantly older than women without a *BRCA1* mutation (43.6 vs. 34.4 years; *P* = 0.007), more likely to be of Ashkenazi Jewish descent (32 % vs. 8 %; *P* = 0.04), to be parous (77.3 % vs. 33.3 %; *P* = 0.003), and to have had a prophylactic mastectomy (41 % vs. 0 %; *P* <0.001). *BRCA1* mutation carriers were also more likely than non-carriers to have undergone a prophylactic salpingo-oophorectomy (59 % vs. 3 %; *P* < 0.001), and consequently, a greater proportion were postmenopausal (59 % vs. 11 %; *P* < 0.001) and had used hormone replacement therapy (HRT) (32 % vs. 8 %; *P =* 0.03). The two groups were similar in terms of breastfeeding, age at menarche, oral contraceptive (OC) use, smoking status, alcohol consumption, and BMI (*P* ≥ 0.20).

**Table 1:** Characteristics of all study participants and stratified by *BRCA1* mutation status

Characteristic	All (n = 58)	*BRCA1* ^+/+^ (n = 36)	*BRCA1* ^+/−^ (n = 22)	*P*
Age (years), mean (range)	38 (18–62)	34.4 (18–62)	43.6 (27–62)	0.007
Ethnicity, *n* (%)				
Other white	34 (59 %)	20 (56 %)	14 (64 %)	0.04
Ashkenazi Jewish	10 (17 %)	3 (8 %)	7 (32 %)	
Hispanic	3 (5 %)	3 (8 %)	0 (0 %)	
East Asian	7 (12 %)	6 (17 %)	1 (4 %)	
South Asian	4 (7 %)	4 (11 %)	0 (0 %)	
Parous, ever, *n* (%)	29 (50 %)	12 (33.3 %)	17 (77.3 %)	0.003
Breastfeeding, ever, *n* (%)^a^	23 (79 %)	10 (83 %)	13 (76 %)	1.00
Age at menarche (years), mean (SD)	12.4 (1.4)	12.2 (1.2)	12.7 (1.7)	0.20
Postmenopausal, *n* (%)	17 (29 %)	4 (11 %)	13 (59 %)	<0.001
Current oral contraceptive use, yes, *n* (%)	6 (10 %)	4 (11 %)	2 (9 %)	1.00
Current smoking status, yes, *n* (%)	1 (2 %)	0 (0 %)	1 (5 %)	0.40
Current alcohol consumption, yes, *n* (%)	51 (88 %)	31 (86 %)	20 (91 %)	0.70
Prophylactic bilateral mastectomy, yes, *n* (%)	9 (16 %)	0 (0 %)	9 (41 %)	<0.001
Prophylactic oophorectomy, yes, *n* (%)	14 (24 %)	1 (3 %)	13 (59 %)	<0.001
Current hormone replacement therapy, yes, *n* (%)	10 (17 %)	3 (8 %)	7 (32 %)	0.03
BMI, kg/m^2^ (SD)	24.2 (5)	24.6 (5)	23.5 (4)	0.39
*BRCA1* mRNA expression, mean (95 % CI)	164.3 (155, 173.5)	175.1 (163.4, 187)	146.7 (134.2, 159)	0.002

^a^Breastfeeding among parous women. *n* number, *SD* standard deviation, *BMI* body mass index defined as mass in (kg) divided by height squared in (m^2^), *CI* confidence interval

### *BRCA1* mutation status significantly contributes to lower overall *BRCA1* expression levels

**Fig. 1:**
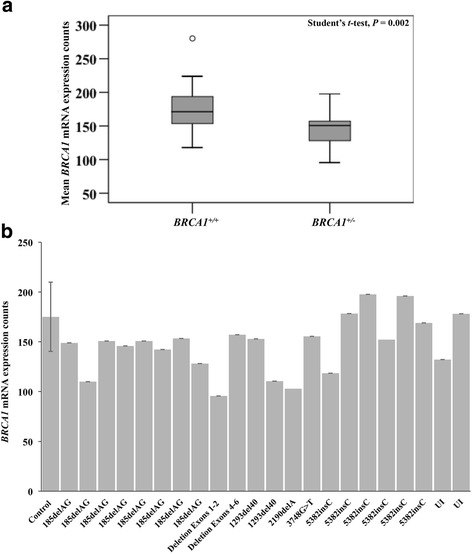
*BRCA1* mRNA expression levels are mutation specific. **a** Box plot analysis of mean *BRCA1* mRNA expression counts in 22 *BRCA1* mutation carriers compared to 36 non-carriers, (146.7 vs. 175.1, *P* = 0.002, respectively). **b** Distribution of *BRCA1* mRNA expression across *BRCA1* mutation carriers compared to non-carriers (i.e., control). *Control* denotes the mean *BRCA1* mRNA expression levels across 36 participants with wild-type *BRCA1* gene status. *Error bars* represent the standard deviation of mean *BRCA1* mRNA expression counts. *UI* mutation is unidentified

The nCounter Analysis System allows the direct measurement of the number of RNA transcripts of the BRCA1 gene, herein, expressed as counts. In the univariate analysis, *BRCA1* mutation carriers had significantly lower mean *BRCA1* mRNA expression compared to non-carriers (146.7 counts vs. 175.1 counts; *P* = 0.002) (Fig. 1, Table 1 and Additional file 1: Table S1). As *BRCA1* mRNA expression levels had a normal distribution as determined by the Shapiro-Wilk test of normality, we employed linear regression modeling to investigate factors associated with *BRCA1* expression (Tables 2 and 3 and Additional file 2: Table S2). We found that mutation status, parity, breastfeeding, menopause, and oophorectomy were each significantly correlated with lower *BRCA1* mRNA levels (*P* ≤ 0.02) (Table 3). Although not significant, age, age at menarche, current OC use and smoking status were negatively associated with *BRCA1* mRNA counts while current alcohol consumption and HRT use were positively associated with *BRCA1* mRNA counts.

**Table 2:** Correlation between reproductive or lifestyle factors and *BRCA1* mRNA expression

Variable	Unstandardized B (95 % CI)	*P* value
Age	−0.63 (−1.3, 0.07)	0.08
Parity (ever)	−22.3 (−40, −4.8)	0.01
Breastfeeding (ever)	−12 (−22, −3)	0.009
Age at menarche	−3.7 (−10.2, 2.8)	0.30
Menopause	−23.3 (−43, −4)	0.02
Current oral contraceptive use	−2.5 (−33, 30)	0.90
Current smoking status	−9.3 (−45, 26)	0.60
Current alcohol consumption	0.115 (−28, 28)	1.00
Mastectomy	−15.3 (−41, 10)	0.23
Oophorectomy	−24 (−45, −3.4)	0.02
Current hormone replacement therapy use	0.5 (−11.8, 12.8)	0.93
Body mass index	0.445 (−1.5, 2.3)	0.65
Mutation status	−28.5 (−46, −11)	0.002

Data are provided as linear regression coefficient, Unstandardized B coefficient and 95 % confidence intervals. *P* value < 0.05 denotes statistical significance

**Table 3:** The mutation status is the most significant contributor to reduced BRCA1 mRNA levels after adjusting for parity, breastfeeding, and menopause

Model	B Coefficient (95 % CI)	*P* value
Constant	179 (128.4, 207.5)	*<*0.001
Mutation status	−22.5 (−43.3, −1.7)	0.04
Pregnancy	3.5 (−28, 35)	0.80
Breastfeeding	−9.3 (−24, 5.6)	0.20
Menopause	−3.5 (−28, 20)	0.70

The model of best-fit incorporated factors significantly contributing to reduced *BRCA1* expression levels by linear regression. Covariates were selected based on univariate analyses showing statistically significant correlations with *BRCA1* mRNA levels. The mutation status remains the most significant contributor to lower *BRCA1* mRNA levels after adjusting for parity, breastfeeding, and menopause (*P* = 0.04). *P* value denotes the contribution of each covariate to the linear regression model

As *BRCA1* mutation status was the most significant contributor to reduced *BRCA1* mRNA expression counts in the univariate analysis, we also evaluated this relationship after adjusting for parity, breastfeeding, menopause, and oophorectomy. Covariates were selected based on the univariate analysis showing statistically significant correlations with *BRCA1* mRNA counts (Table 2 and Additional file 2: Table S2). For the final regression model, parity, breastfeeding, menopause and mutation status were included in the analysis. As oophorectomy results in menopause, it was excluded from the analysis to prevent over-adjustment. Multivariate modeling demonstrated that carrying a *BRCA1* mutation remained the most significant predictor of *BRCA1* mRNA levels (Table 3). After adjusting for other important covariates, women with a *BRCA1* mutation had 22.5 lower counts of *BRCA1* mRNA levels compared to non-carriers (*P* = 0.04). These data suggest that *BRCA1* mutation status alone predicts *BRCA1* mRNA expression levels.

### *BRCA1* mRNA expression levels are mutation specific

Next we characterized *BRCA1* mRNA expression by mutation position and type (Table 4 and Fig. 1). Germline mutations in *BRCA1* may be located within any of the 22 exons of the gene, with the majority of known pathogenic mutations generating premature termination codons (PTCs). mRNA transcripts with PTCs are typically degraded by a mechanism called nonsense-mediated mRNA decay (NMD) in order to prevent the synthesis of potentially harmful truncated protein products [44]. The decay of mutant *BRCA1* mRNA has been shown to result in a 1.5 to 5-fold decrease in mRNA abundance; however, PTCs located very close to the translation initiation codon in exon 2 (185delAG) or in the last exon (i.e., 5382insC) may escape NMD [44, 45]. Table 4 outlines the mutation type and location within the gene and whether the mutation type results in NMD [28, 44, 46]. There were seven distinct *BRCA1* mutations among the 22 study participants with a known *BRCA1* mutation. The mutation type for two of the 22 mutation carriers was unknown. Fourteen of the 22 mutation carriers shared one of two specific mutations: (1) 185delAG or (2) 5382insC, both of which are likely to escape nonsense-mediated mRNA decay. Three distinct mutations, including, 2190delA, 1293del40, and 3748G > T, were suggested to undergo NMD and had lower *BRCA1* mRNA expression counts compared to mutations that escape NMD, though not statistically significant (mean counts of 130.5 vs. 153 counts, respectively, *P* = 0.15). Large rearrangement mutations, including deletion exons 1–2 and deletion exons 4–6, had different expression levels at 95.56 counts and 157.12 counts, respectively. Interestingly, participants with mutations that do not undergo NMD had variable expression levels, suggesting that additional factors might regulate *BRCA1* mRNA levels (Fig. 1b). For example, 185delAG *BRCA1* mutation carriers had expression levels ranging between 109.9 and 153.3 counts. Similarly, 5382insC *BRCA1* mutation carriers had expression levels ranging between 118.4 and 195.9 counts. Overall, the data suggest *BRCA1* basal mRNA expression levels may be mutation specific.

**Table 4:** Overview of *BRCA1* mutation type and association with nonsense-mediated decay

Study ID	Age	*BRCA1* mutation	Mutation type	Exon	^a^Nonsense-mediated decay (NMD)
1	33	Deletion Exon 1-2	FS	1–2	Unknown
2	54	185delAG	FS	2	-
3	31	185delAG	FS	2	-
4	27	185delAG	FS	2	-
5	36	185delAG	FS	2	-
6	58	185delAG	FS	2	-
7	58	185delAG	FS	2	-
8	44	185delAG	FS	2	-
9	50	185delAG	FS	2	-
10	35	Deletion Exon 4-6	FS	4–6	Unknown
11	54	2190delA	FS	11	+
12	60	1293del40	FS	11	+
13	33	3748G > T	NS	10	+
14	51	1293del40	FS	11	+
15	33	5382insC	FS	20	-
16	38	5382insC	FS	20	-
17	62	5382insC	FS	20	-
18	52	5382insC	FS	20	-
19	35	5382insC	FS	20	-
20	38	5382insC	FS	20	-
21	41	UI			
22	37	UI			

^a^Nonsense-mediated decay (NMD) status was based on experimental investigations by Liu *et al.* and Perrin-Vidoz *et al.* [44–46] and other reports [19–28]. *UI* mutation is unidentified, *FS* frameshift mutation, *NS* Nonsense mutation, *NMD (+)* nonsense-mediated decay is present, *NMD (-)* nonsense-mediated decay is absent, *NMD (Unknown)* functional contribution of NMD to mRNA expression levels in these mutations remains to be explored

**Fig. 2:**
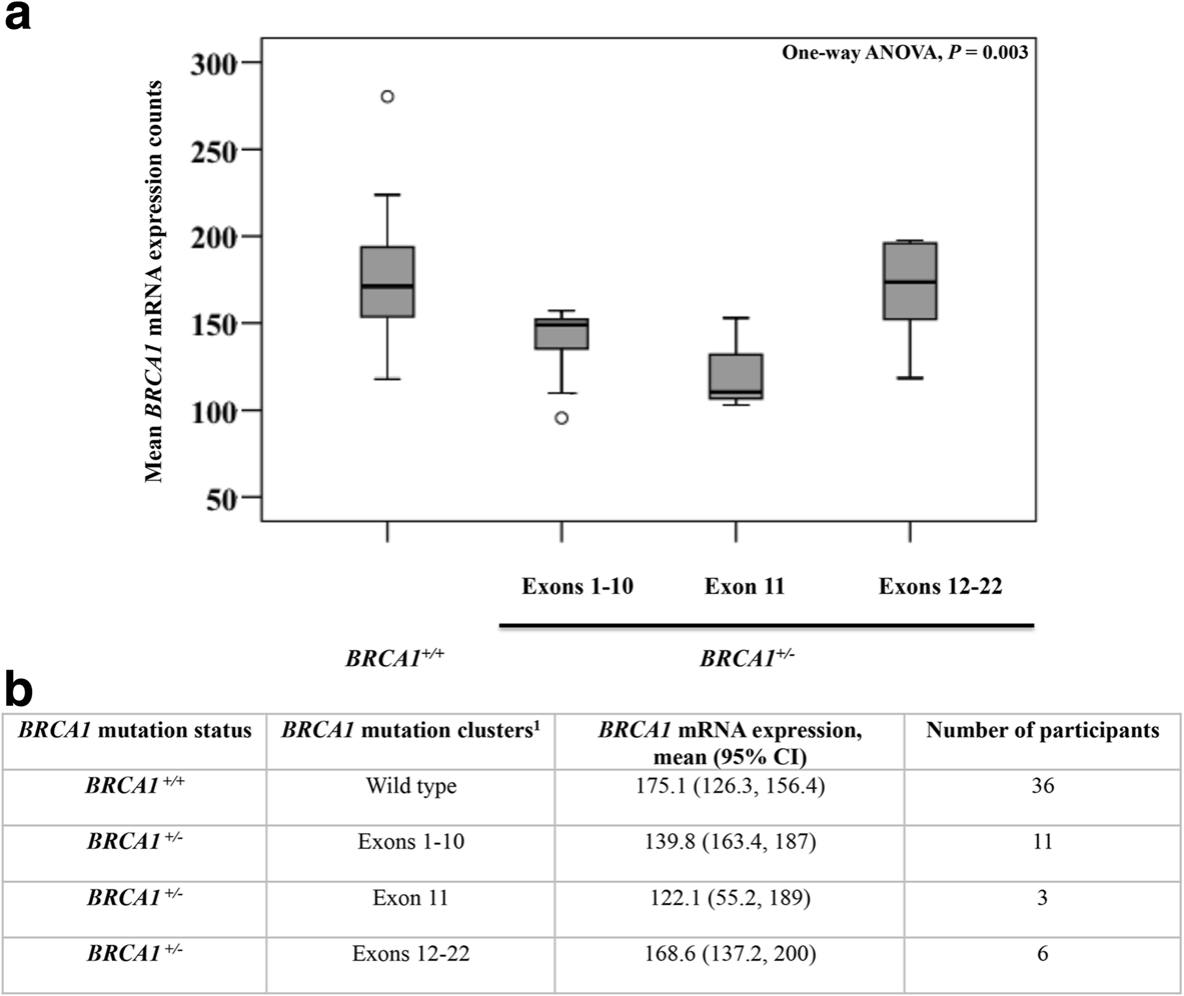
Stratification of mean *BRCA1* mRNA expression counts by *BRCA1* mutation clusters associated with differential risk for breast and/or ovarian cancers. **a** Box plot distribution of mean BRCA1 mRNA counts by location of mutation compared to non-carriers. *ANOVA* analysis of variance. **b** ^1^Mutations were sub-classified into three clusters: mutations in the 5′ terminal (Exons 1-10) and 3′ terminal (Exons 12-22) of exon 11 include three previously identified breast cancer cluster regions (BCCRs) proposed to have increased risk for breast vs. ovarian cancer. Mutations within exon 11 were shown to have increased risk for ovarian vs. breast cancer [28]

Recently, Rebbeck et al. [28] reported that mutations in the 5′ and 3′ regions of *BRCA1* were associated with an increased risk of breast cancer, while mutations in exon 11 were associated with an increased risk of ovarian vs. breast cancer. To determine if these specific mutation clusters were associated with differential *BRCA1* mRNA expression in our cohort, mutations were pooled into three groups: group 1 containing mutations in exons 1–10; group 2 containing mutations in exon 11; and group 3 containing mutations in exons 12–22 (Fig. 2a, b) [28]. Mutations within exon 11 had lower mRNA expression levels (122.1 counts) compared to mutations within the 5′ (138.9 counts) and 3′ (168.6 counts) regions of the *BRCA1* gene (*P* = 0.003) (Fig. 2a, b).

### *BRCA1* mutation carriers have similar gene expression profiles

**Fig. 3:**
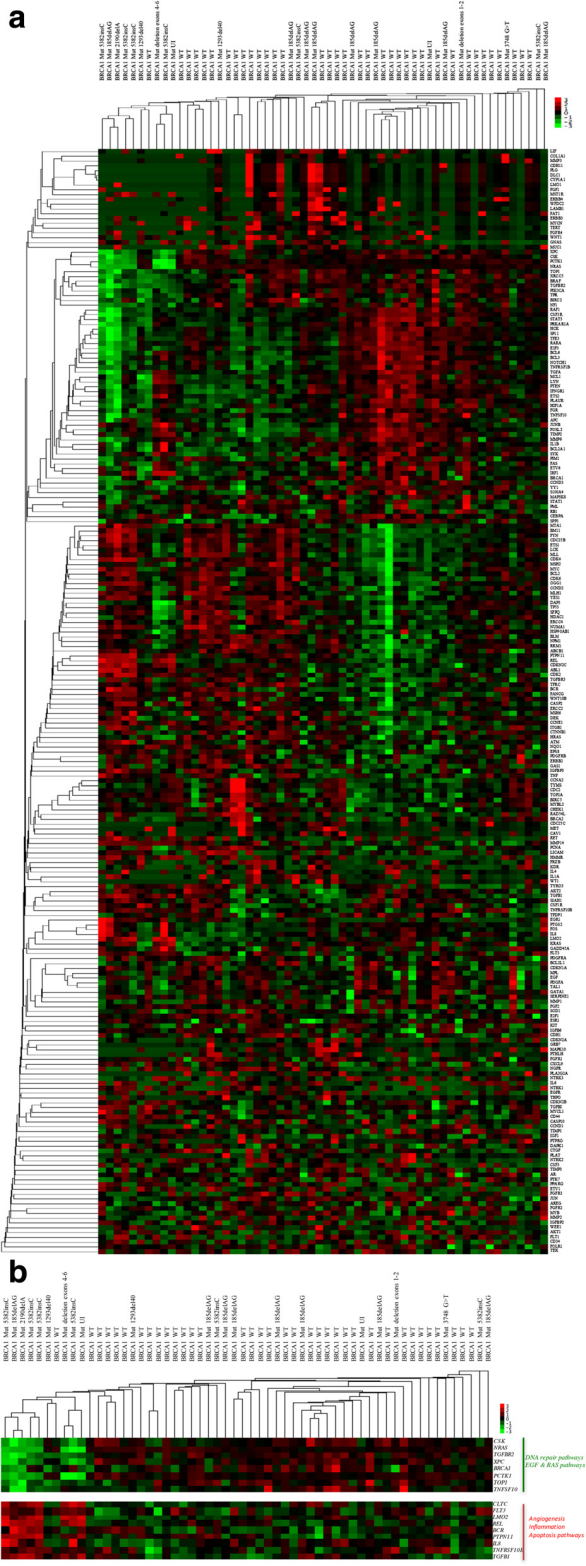
Comparison of gene expression profiles of *BRCA1* mutation carriers (n = 22) and non-carriers (n = 36). **a** *Heat map* showing unsupervised hierarchical clustering of 236 genes from the Nanostring Cancer Reference gene panel across *BRCA1* mutation carriers and non-carriers. *BRCA1 Mut*: *BRCA1* mutation carrier status and type; *BRCA1 WT*: *BRCA1* wild-type gene status. **b** Unsupervised hierarchal clustering of gene expression profiles of freshly isolated blood leukocytes from *BRCA1* mutation carriers and non-carriers, showing samples cluster by *BRCA1* mutation status: 9/22 *BRCA1* mutation carriers share similar gene expression profiles and cluster more closely together. *Heat map* shows the top differentially expressed genes and corresponding gene pathway enrichment analysis; *green* shows relatively under expressed genes and pathways, respectively; *red* shows relatively over-expressed genes and pathways, respectively

To further characterize the gene expression profiles of freshly isolated blood leukocytes from women with and without a *BRCA1* mutation, unsupervised clustering, using a Pearson-centered correlation metric with centroid linkage rules of the normalized genes, was performed across all the samples. Interestingly, the samples tended to cluster based on *BRCA1* mutation status (Fig. 3). Out of 236 genes included in the nCounter® GX Human Cancer Reference Kit, we identified eight genes to be significantly downregulated in carriers (Table 5), including *BRCA1, CSK*, *NRAS*, *PCTK1*, *TGFBR2*, *TNFSF10*, *TOP1*, and *XPC*. Nine genes were significantly upregulated in carriers, including *BCR*, *CLTC*, *FLT3*, *IL8*, *LMO2*, *PTPN11*, *REL*, *TGFB1* and *TNFRSF10B* at a Benjamini-Hochberg false discovery rate <0.05 using the *t* test (Table 6). PathDIP (http://ophid.utoronto.ca/pathDIP) was used for pathway enrichment analysis and identified pathway associations for 94 % of genes. PathDIP analysis showed significant enrichment for cancer pathways (especially colorectal and pancreatic cancers), Toll-like receptor signaling, RAS signaling, and numerous other pathways (Additional file 3: Table S3) (*P* < 0.0000001). Upregulated genes were most enriched for integrins in angiogenesis, advanced glycation end products and receptor AGE-RAGE in inflammation, and apoptosis pathways (*P* < 0.0000001). Downregulated genes were most enriched for EGF, RAS, and DNA repair pathways, and for hearing and vision proteins (*P* < 0.0001).

**Table 5:** A summary of genes significantly downregulated in samples of *BRCA1* mutation carriers compared to non-carriers (Benjamini-Hochberg false discovery rate < 0.05 using the *t*-test)

Gene	Gene name	*P* value
*CSK*	c-Src tyrosine kinase	0.000
*NRAS*	Neuroblastoma RAS viral (v-ras) oncogene homolog	0.000
*TGFBR2*	Transforming growth factor, beta receptor II	0.001
*XPC*	Xeroderma pigmentosum, complementation group C	0.001
*BRCA1*	Breast cancer 1, early onset	0.002
*PCTK1/CDK16*	Cyclin-dependent kinase 16	0.008
*TOP1*	Topoisomerase (DNA) I	0.008
*TNFSF10*	Tumor necrosis factor (ligand) superfamily, member 10	0.009

**Table 6:** A summary of genes significantly upregulated in samples of *BRCA1* mutation carriers compared to non-carriers (Benjamini-Hochberg false discovery rate < 0.05 using the *t* test)

Gene	Gene name	*P* value
*CLTC*	Clathrin, heavy chain (Hc)	0.000
*FLT3*	Fms-related tyrosine kinase 3	0.002
*LMO2*	LIM domain only 2	0.002
*REL*	V-Rel avian reticuloendotheliosis viral oncogene homolog	0.002
*BCR*	Breakpoint cluster region	0.004
*PTPN11*	Protein tyrosine phosphatase, non-receptor type 11	0.004
*TNFRSF10B*	Tumor necrosis factor superfamily, member 10B	0.007
*IL8*	Interleukin 8	0.009
*TGFB1*	Transforming growth factor, beta 1	0.009

## Discussion

The goal of the current study was to evaluate whether *BRCA1* mRNA expression levels are reduced in the leukocytes of women with a *BRCA1* mutation. We found that carrying a *BRCA1* mutation was the most significant predictor of *BRCA1* mRNA levels. *BRCA1* mRNA levels were significantly lower in mutation carriers compared to non-carriers (146.7 counts vs. 175.1 counts; *P* = 0.002). Furthermore, mutations within exon 11 were associated with lower mRNA expression levels compared to mutations within the 5′ and 3′ regions of the *BRCA1* gene (122 counts vs. 138 and 167 counts, respectively; *P* = 0.003). In addition, 17 other genes in a panel of 236 genes, some of which have been previously shown to be involved in breast and ovarian carcinogenesis, were also differentially expressed between the two groups of women. Overall, these findings support the concept of *BRCA1* haploinsufficiency, whereby a dosage-dependent effect of the *BRCA1* gene is associated with molecular alterations at the transcriptional level. Reduced *BRCA1* mRNA expression levels were previously reported in *BRCA1*-associated cancer [38]. To our knowledge, this is the first study to identify lower *BRCA1* expression in leukocytes from healthy, unaffected *BRCA1* mutation carriers. Whether lower *BRCA1* transcript levels translate to changes in protein levels or to carcinogenesis remains to be determined.

Emerging evidence from epidemiologic studies, from human tissue culture model systems and from murine studies supports a continuum of tumor suppression where *BRCA1* expression levels might be tightly correlated with function [9]. Recently, Pathania et al. reported that human breast epithelial and skin fibroblast cells from *BRCA1* mutation carriers had lower BRCA1 protein levels that were associated with haploinsufficiency for stalled replication fork repair/replication stress and conditional haploinsufficiency for HR-DSB [22]. Under concurrent forms of stress, such as UV and IR that require BRCA1 mediated HR-DSB, the pool of available BRCA1 is not sufficient to repair all the damage, thereby leading to the accumulation of small genomic aberrations. When these aberrations reach a threshold above which the damage is irreparable, genomic instability ensues and drives malignant transformation [8]. Consequently, identifying *BRCA1* mutations that result in low basal expression levels might help stratify cancer risk. More recently, Sedic et al. showed that *BRCA1* mutation carriers exhibit cell-type-specific haploinsufficiency for genomic instability, whereby human mammary epithelial cells from *BRCA1* mutation carriers had shorter telomeres that contributed to premature senescence compared to non-carriers [23]. If these in vitro findings translate to mammary epithelial cells having a reduced life span in vivo, then additional genomic alterations are required to bypass premature senescence. Whether *BRCA1* expression levels can predict cells that are at increased risk of escaping senescence remains to be determined.

A number of epidemiologic reports suggest tumor type predisposition differs according to the position of the *BRCA1* mutation [27–29]. In an analysis of 60 families with a history of breast or ovarian cancer, Gayther et al. showed that *BRCA1* mutations mapping up to and including exon 12 were linked to a higher ratio of ovarian to breast cancers compared to mutations mapping to the C-terminal portion of the *BRCA1* gene [29]. Moreover, using genotype-phenotype correlations in 356 families with pathogenic *BRCA1* mutations, Thompson et al. showed that the ovarian to breast cancer ratio was higher with mutations in the central region of the *BRCA1* gene (nucleotides 2401-4190; predominantly within exon 11) [27]. In a systemic analysis of 19,581 carriers of *BRCA1* mutations, Rebbeck et al. identified that mutation clusters mapped to the 5′ (BCCR1, c.179 to c.505) and 3′ (BCCR2, c.4328 to c.4945 and BCCR2′, c.5261 to c.5563) regions of *BRCA1* were associated with increased breast cancer risk, while mutations mapped to exon 11 were associated with increased ovarian cancer risk [28].

In the current study, we showed that mutations within exon 11 had the lowest mRNA expression levels compared to mutations within the 5′ and 3′ regions of the *BRCA1* gene, with nonsense-mediated decay being the most likely mechanism mediating mutation-dependent reduced transcript level readout. It remains to be determined how mutation-dependent modulation of *BRCA1* expression levels affects the different functions of BRCA1, i.e., which mutations confer a dominant negative role compared to mutations that retain partial wild-type BRCA1 function, whether functions involving domains within exon 11 are more relevant to ovarian epithelium function and whether those within domains of the 3′ and 5′ regions more relevant to breast epithelium functioning [29].

Conditional *Brca1* mouse models have also provided insight into functional correlates of different *BRCA1* mutations. Mouse strains carrying loss of *Brca1* and heterozygous *p53* mutations are conditionally targeted to mammary epithelial cells using Cre-lox system. Shakya et al. showed that introduction of a *brca1* mutation I26A, which impairs E3 ubiquitin ligase activity but maintains the interaction with Bard1, does not lead to tumor formation; whereas mutation of the BRCT domain (S1598F) that disrupts phosphoprotein binding resulted in a high rate of tumor formation [26]. Drost et al. demonstrated that the *Brca1* (C61G) missense mutation, which impairs BRCA1/BARD1 heterodimerization and ubiquitin ligase activity, resulted in mammary tumors that were resistant to cisplatin and PARP inhibitors compared to *Brca1* null mice [25]. Overall, these studies suggest that all *BRCA1* mutations are not equivalent in their tumorigenic potential, and consequently, cancer risk assessment might be mutation specific.

The present study also highlights *BRCA1* mutation status as a significant classifier based on global gene expression profiling. Pathway enrichment analysis revealed gene expression alterations that are tissue specific, including genes that mediate acute/chronic leukemia. Interestingly, enrichment of genes involved in Toll-like receptor signaling and IL-2-mediated signaling in *BRCA1* mutation carriers, suggests that changes in the immune microenvironment might occur early on in heterozygous cells and may be useful for targeted prevention strategies. Furthermore, downregulated genes were enriched in DNA damage response pathways, thereby supporting the growing evidence for *BRCA1* haploinsufficiency in DNA damage repair as a potential early tumor predisposing event.

Strengths of the current study include the use of RNA from blood leukocytes that was stabilized within 30 minutes of collection, resulting in high quality RNA (mean RNA integrity number, RIN = 8.7), and the use of the NanoString nCounter Analysis System to quantify mRNA expression, which helps to achieve high validity, reproducibility, and sensitivity [42]. The primary limitation of our study is that the *BRCA1* expression analysis was not allele-specific, i.e., the lower mRNA counts in leukocytes of *BRCA1* mutation carriers extend beyond nonsense-mediated decay of the mutant allele to include mechanisms, such as microRNA-mediated regulation of expression levels of wild-type or mutant-type alleles, and altered expression levels of regulators of *BRCA1* mRNA levels in *BRCA1* mutation carriers vs. non-carriers. Future studies using RNA sequencing, which can discriminate between levels of the wild-type and mutant allele are warranted. Other limitations include the relatively small sample size that allowed for the evaluation of a narrow spectrum of *BRCA1* mutations and their effects on *BRCA1* gene expression levels.

Collectively, these data support the concept of *BRCA1* haploinsufficiency, whereby *BRCA1* heterozygous cells have lower *BRCA1* mRNA expression levels. Since the evaluation of BRCA1 protein levels as a surrogate marker of *BRCA1* haploinsufficiency posits a challenge, especially in freshly isolated leukocytes, our study highlights the role of using *BRCA1* mRNA levels as an indicator of prospective BRCA1 functional levels. Factors that increase *BRCA1* levels to a normal level might restore BRCA1 function. We recently demonstrated that daily oral supplementation with 3,3′-diindolylmethane (i.e., DIM) for 4–6 weeks resulted in a significant 34 % increase in *BRCA1* mRNA expression in leukocytes from women with a *BRCA1* mutation [47]. Elsewhere, sedentary behavior was associated with significantly lower *BRCA1* mRNA levels in women with and without a mutation [48]. These studies provide important mechanistic insight into how a lifestyle factor may mediate cancer risk in this high-risk population.

## Conclusions

In summary, our findings suggest that *BRCA1* mutation status is a significant predictor of lower *BRCA1* mRNA levels in peripheral blood leukocytes. In turn, this may provide a feasible tool whereby modulation of *BRCA1* levels through different interventions can be monitored in the clinical setting or can stratify risk. The possibility of mitigating the effect of an inherited deleterious *BRCA1* mutation by increasing the physiologic expression of the gene and normalizing protein levels represents a clinically important paradigm shift in the prevention strategies available to these high-risk women.

## Abbreviations

AGE-RAGE, advanced glycation end products-receptor for advanced glycation end products; ANOVA, analysis of variance; BCCR, breast cancer cluster region; *BRCA1*, Breast cancer 1, early onset; DSB, double strand break; HR, homologous recombination; HR-DSB, homologous recombination mediated repair of double-stranded DNA breaks; HRT, hormone replacement therapy; mRNA, messenger RNA; NMD, nonsense mediated decay; PTC, premature termination codon; WCRI, Women’s College Research Institute

## Additional files

Additional file 1: Table S1. Distribution of mean *BRCA1* mRNA expression counts in *BRCA1* mutation carriers and non-carriers. (DOC 31 kb) Additional file 2: Table S2.
*BRCA1* mutation status is a predictor of *BRCA1* mRNA expression. (DOC 32 kb) Additional file 3: Table S3. Pathway enrichment analysis of genes differentially expressed between *BRCA1* mutation carriers and non-carriers. (XLS 205 kb)
