# Supplementary material for: Reduced BRCA1 transcript levels in freshly isolated blood leukocytes from BRCA1 mutation carriers is mutation specific
Source: Breast Cancer Res. 2016 Aug 17;18:87. doi: 10.1186/s13058-016-0739-8 (PMC4989508; doi:10.1186/s13058-016-0739-8)
Supplement: Additional file 1: Table S1. — Distribution of mean BRCA1 mRNA expression counts in BRCA1 mutation carriers and non-carriers. (DOC 31 kb) [file 13058_2016_739_MOESM1_ESM.doc]

**Table S1. Distribution of mean BRCA1 mRNA expression counts in BRCA1** mutation carriers and non-carriers

|  |  | ***BRCA1 ^+/+^, n=36*** | ***BRCA1 ^+/-^, n=22*** |
| --- | --- | --- | --- |
|  | **Mean** | 175.1 | 146.7 |
|  | **Median** | 171.1 | 150.8 |
|  | **Std. Deviation** | 34.8 | 28 |
|  | **Minimum** | 118 | 96 |
|  | **Maximum** | 280 | 198 |
| **Percentiles** | **25th** | 151 | 125.7 |
|  | **50th** | 171.1 | 150.8 |
|  | **75th** | 194.8 | 160 |
