# Supplementary material for: Reduced BRCA1 transcript levels in freshly isolated blood leukocytes from BRCA1 mutation carriers is mutation specific
Source: Breast Cancer Res. 2016 Aug 17;18:87. doi: 10.1186/s13058-016-0739-8 (PMC4989508; doi:10.1186/s13058-016-0739-8)
Supplement: Additional file 2: Table S2. — BRCA1 mutation status is a predictor of BRCA1 mRNA expression. (DOC 32 kb) [file 13058_2016_739_MOESM2_ESM.doc]

**Table S2. BRCA1 mutation status is a predictor of BRCA1** mRNA expression

| **Covariate with mutation status** | **Adjusted B-Coefficient**  **(95% CI)** | ***P value*** | **R^2^*, Adjusted* R^2^*, P’*** |
| --- | --- | --- | --- |
| Parity | -23 (-42, -3.5) | 0.02 | 0.19, 0.16, 0.003 |
| Breastfeeding | -23 (-41, -5) | 0.01 | 0.21, 0.18, 0.001 |
| Menopause | -23 (-44, -3) | 0.03 | 0.17, 0.14, 0.005 |
| Oopherectomy | -25 (-48, -2) | 0.03 | 0.16, 0.13, 0.008 |
| Parity, Breastfeeding, Menopause | -22.5 (-43.3, -1.7) | 0.04 | 0.21, 0.15, 0.012 |

Since mutation status was the most significant contributor to *BRCA1* mRNA expression levels by univariate analysis, we sought to determine the contribution of the mutation status to *BRCA1* mRNA expression after adjusting for the following covariates: parity, breastfeeding, menopause, and oophorectomy. Covariates were selected based on univariate analyses showing statistically significant correlations with *BRCA1* mRNA levels. Regardless of the covariates used in the analysis, the mutation status remains the most significant contributor to reduced *BRCA1* mRNA expression levels. Parity, breastfeeding, menopause and mutation status were included in the final regression model. Since oophorectomy is a preventative measure for *BRCA1* mutation carriers and results in menopause, it was excluded from the analysis to prevent over-adjustment. Abbreviations: Unstandardized B Coefficient and 95% confidence intervals; *P* value denotes the contribution of the mutation status to reduced *BRCA1* mRNA expression levels after adjusting for the various cofactors. R^2^, Adjusted R^2^, and *P’* denote the power of the suggested model to predict *BRCA1* mRNA expression levels.
